# Data insights from a Moroccan phytochemical database (MPDB) derived from aromatic & medicinal plants

**DOI:** 10.6026/973206300191217

**Published:** 2023-12-31

**Authors:** Elkhattabi Lamiae, Zouhdi Salwa, Mousstead Fairouz, Karima Mohtadi, Hassan Fougrach, Wadi Badri, Hassan Taki, Anass Kettani, Mohammed Talbi, Rachid SAILE

**Affiliations:** 1 Laboratory of Biology and Health, Faculty of Sciences Ben M'Sik, Hassan II University of Casablanca, Morocco; 2 Laboratory of ecology and environment, Faculty of Sciences Ben M'Sik, Hassan II University of Casablanca, Morocco; 3 Laboratoire de Chimie Analytique et Moléculaire LCAM faculté des sciences Ben Msik, Hassan II University of Casablanca, Morocco

**Keywords:** aromatic and Moroccan plants, phytochemical database, virtual screening

## Abstract

The geographical location of Morocco and the diversity of its topography ensure a high variability of climate conditions, ranging from humid to Saharan, and extending through subhumid, arid, and semi-arid stages. This variability offers a high floristic diversity, while the medical use of these phytochemicals has not been fully explored. Advanced computer-aided drug discovery utilizes chemical biology to accelerate the study of phytochemicals at the molecular level and discover novel therapeutic pathways. Currently, there is no online resource for phytochemicals in Morocco. Therefore, it is of interest to describe the Moroccan Phytochemicals Database (MPDB), accessible, featuring over 600 phytochemicals derived from journal articles and other reports. The web interface of the database, which is simple and easy to use, provides each phytochemical's reference, plant sources, 3D structures, and all related information. Furthermore, we provide direct links to commercially available analogs from Mcule. In addition, we provide the results of the first virtual screening against cardiovascular targets. We present these data to facilitate further exploration and exploitation of Morocco's rich phytochemical resources, and to contribute to the global understanding and application of these compounds in the medical and scientific communities.

## Background:

Morocco's extraordinary ecological and plant diversity can be largely attributed to its unique geographical location. The country boasts approximately 4,500 species and subspecies of vascular plants, a remarkable 14.22% of which are medicinal, and 22% are endemic [1]. Moreover, Morocco's diverse ecosystems and the high level of endemism among its animal species distinguish it from other regions. Indeed, Morocco is considered a vital global hotspot for biodiversity, hosting many unique and irreplaceable species found nowhere else [2]. Historically, medicinal plants have been recognized for their significant role in healthcare, providing remedies for various ailments, including cardiovascular diseases, inflammation, and weakened immune systems. Phytochemicals have demonstrated the potential to regulate biological activities, as exemplified by aspirin, which is recognized for its therapeutic properties, including antioxidant potential [3]. The regular consumption of diets rich in phytochemicals, including fruits, vegetables, and whole grains, may reduce the risk of diseases associated with oxidative damage [4]. The wide acceptance and compatibility of phytochemicals with the human body, their unique structural features, and minimal adverse effects contribute to their substantial usage [5]. Furthermore, a significant proportion of pharmaceutical drugs are derived from plant extracts [6]. The diverse structures and biological activities of phytochemicals captivate both laboratory and computational scientists, serving as blueprints for developing small molecules and drugs; they are recognized as privileged structures to interact with protein drug targets. Their unique characteristics and structural diversity continue to amaze scientists in the development of natural inspired medicines [7]. Computational methodologies have been incorporated into the drug discovery pipeline to aid in the identification and elucidation of these unique compounds, focusing on identifying molecular patterns and structures to guide combinatorial design and target selectivity [8]. Over the years, disciplines such as chemo-informatics and bioinformatics have made significant contributions to natural product-based drug discovery [9]. The integration of computational strategies, including virtual screening and machine learning, has propelled research on phytochemicals, improving our understanding of these compounds. Consequently, these advances in computational approaches have highlighted the potential of phytochemicals as valuable sources of modern drugs, paving the way for the development of new and effective treatments [10]. Hence, it is important to develop a free online database dedicated to Moroccan medicinal and aromatic plants, serving as a comprehensive resource for drug discovery studies utilizing Moroccan phytochemicals. This database will enable users and researchers to identify phytochemicals from Moroccan plants and access their 3D structures, crucial for advanced molecular modelling and virtual screening. Therefore, it is of interest to optimize the exploration of Moroccan phytochemicals, fostering collaboration and highlighting the potential therapeutic benefits of Morocco's plant biodiversity.

## Methodology:

## Server and database design:

**Figure 2:**
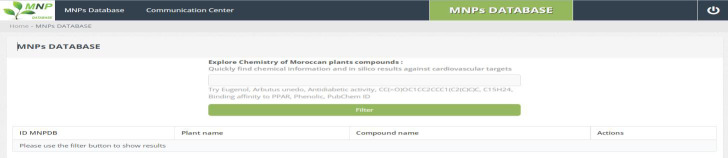
A snapshot of the main menu page. The search for phytochemicals can be conducted using various keywords, as shown.

The MPDB is a database platform built on the open-source database management software MySQL (http://www.mysql.com). We developed the platform's website using PHP (http://www.php.net/) for server-side scripting and JavaScript (http://www.javascript.com/) along with Ajax for client-side scripting, facilitating the creation of user-friendly interfaces. To ensure platform security, we utilized OpenSSL (https://www.openssl.org/) to generate a security certificate, encrypting all communications between users and the application server. Our platform is accessible via the domain name https://mpdb.org/ and can be accessed using any web browser. For managing chemical information within the database, we employed the Open Babel toolbox (http://openbabel.org), which offers a comprehensive suite of tools for chemical data processing. The database schema as shown in Figure 1 (See PDF) is primarily focused on the plant name, phytocompounds, and references tables. These tables store information related to compounds, their sources, and their recorded uses. The sources table refers to the plants from which a given compound was isolated, while the uses table contains information about any specific activity that has been tested for a given compound (such as antimalarial, anti-diabetic, or other activities). This allows researchers and users to easily search and filter the data to find the information they need (Figure 2). Overall, MPDB is a powerful and user-friendly database platform that makes it easy to access and work with chemical data. Its use of open source software and encryption ensures the security of user data, while its flexible schema and interface make it a valuable tool for chemical researchers and professionals. We rigorously verified the accuracy of our phytocompound dataset through a meticulous manual process. We cross-referenced each phytocompound's chemical formula and SMILES notation with reliable sources and compared our results with the PubChem database. We thoroughly investigated any inconsistencies or errors in our dataset and achieved a high level of confidence in its accuracy and integrity. This meticulous verification process is critical for the success of future research in the Moroccan phytochemicals field, and our dataset is now a reliable resource for such research.

## Compound uploading:

**Figure 3:**
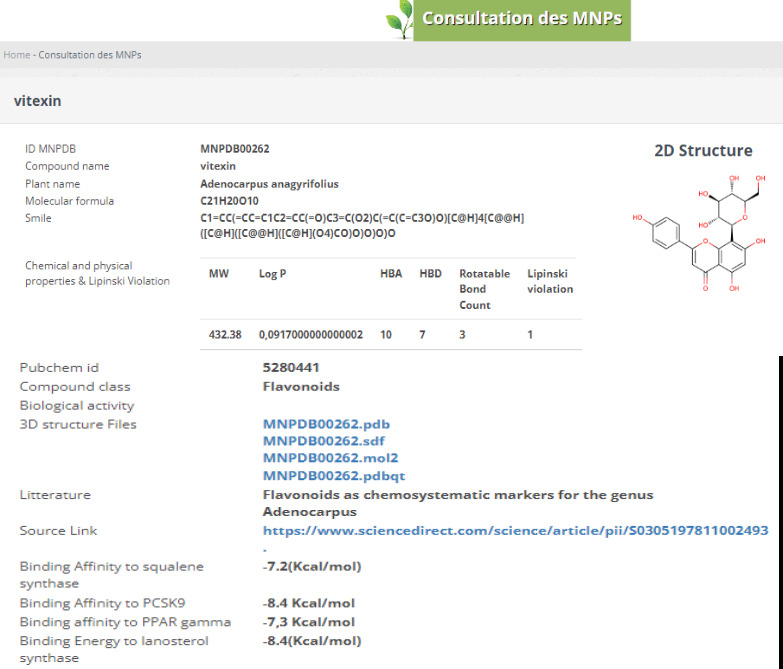
Snapshot of the compound summary page. The interface for the MPDB website is shown, with a screenshot of the part of the compound summary page for entry MNPDB00262.

Compound information was retrieved from journal articles and theses. The name of each plant and compound was extracted, as well as any available information on the compound or plant's activity as shown in Figure 3. However, the SMILES notation for some compounds was not readily available in the publications. To address this, the PubChem database was used to obtain the SMILES and 3D structures for each compound in sdf format. The Open Babel program was then utilized to generate various 3D files such as pdbqt, mol2, and pdb. Additionally, a PubChem ID was added to the extracted data if available. To create 2D structure images of each compound, the Smi2Depict web tool available at http://cdb.ics.uci.edu/cgibin/Smi2DepictWeb.py was used.

## Analogs collection:

To identify analogs for each entry in the MPDB, we utilized the Mcule database (11) and extracted a set of compounds in SMILES format, which we downloaded. We then calculated Tanimoto coefficients or similarity scores using the OpenBabel software (12). A Tanimoto coefficient cut-off of 0.8 or greater was used to identify analogs. We integrated the analog ID along with their respective Mcule links into the MPDB, as shown in Figure 7. This process allowed us to expand the scope of the MPDB by incorporating analogs of the compounds already present in the database. By including analogs, we were able to identify additional potential leads for drug discovery and further broaden the potential applications of the MPDB. The Tanimoto coefficients served as a measure of the similarity between the compounds, allowing us to identify those with the greatest potential for further investigation.

## Referenced database:

The MPDB website provides a comprehensive list of publications where phytocompounds have been identified, including the title and DOI of the journal article or a direct link to the source. Given that the database was constructed through manual extraction of information from journal articles, it was deemed essential to maintain a strong connection between the references and the data obtained from them. This feature enables the website to function as a valuable research tool, specifically dedicated to Moroccan phytochemicals. In case a user discovers a relevant compound, they may seamlessly navigate to the literature available on that specific phytochemical. The website also presents a larger-scale opportunity to explore literature describing Moroccan phytochemicals with a particular activity. This resource simplifies information access and boosts the visibility of related research.. A further advantage of maintaining fully referenced information is the ability to locate compounds within a publication, which can prove challenging at times.

## Phytochemical classification:

The classification of phytochemicals is a useful tool for assessing their diversity. In this study, we used ClassyFire, a hierarchical classification system that groups compounds based on their structural patterns [13].

## Chem-informatics analysis:

Lipinski's rule of five, a widely accepted set of guidelines, has proven to be an effective tool for determining the drug-likeness of a compound. This rule takes into account physicochemical parameters such as molecular weight, hydrogen bond donors and acceptors, and the calculated octanol-water partition coefficient (c log P). In this study, we evaluated the compliance of the compounds in the MPDB with Lipinski's rule.

## Results:

**Figure 4:**
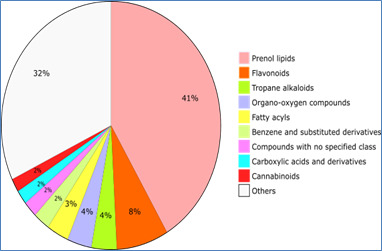
MPDB; classification. Classifications were obtained from ClassyFire.

Phytochemical classification: Prenol lipids stand out as a dominant presence, comprising roughly 41.90% of the total cataloged phytochemical compounds within the MPDB database. These prenol lipids play an indispensable role across a spectrum of biological processes, serving as key players in cell signaling and orchestrating the regulation of diverse metabolic pathways. Within the MPDB dataset, flavonoids command approximately 7.75%, showcasing their prevalence as natural pigments embedded within the plant kingdom. Acknowledged for their robust antioxidant properties and potential health-enhancing attributes, flavonoids hold promise as bioactive compounds with multifaceted applications. The realm of tropane alkaloids, constituting approximately 3.76%, emerges as another intriguing category of phytochemical compounds. Boasting pronounced pharmacological effects, their presence within the MPDB database could captivate researchers engaged in the intricate realm of harnessing nature's offerings for novel drug development. Organo-oxygen compounds contribute approximately 3.64%, while fatty acyls make up around 3.29% of the phytochemical landscape. Benzene and substituted derivatives account for about 2.46%, and compounds with no specified class represent 2.11%. Carboxylic acids and derivatives contribute 1.88%, and cannabinoids make up 1.76%. Lastly, the "Others" category, which includes anthracene, aryltetralin lignans, benzopyrans, cinnamic acids and derivatives, coumarins and derivatives, dibenzyl butane lignans, dihydropyran, epoxides, furanoid lignand, furans, furofurans, indoles and derivatives, lactones, oxanes, peptide-mimetics, phenanthrenes and derivatives, phenol esters, phenol, polycyclic hydrocarbons, pyrans, quinolines and derivatives, saturated hydrocarbons, steroids and steroid derivatives, tetra-hydrofurans, tetralin, and unsaturated hydrocarbons, accounts for 27.51% of the total phytochemical compounds cataloged in the MPDB database. These findings indicate a diverse range of compounds with the potential for various biological activities. The distribution of compound classes can be seen in Figure 4.

**Figure 5:**
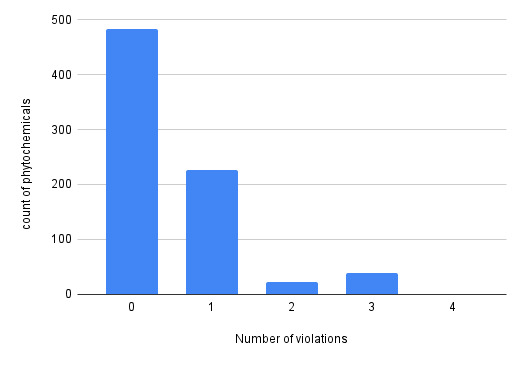
The tabulated compounds in the database with their number of violations of Lipinski's "rule of five.

As illustrated in Figure 5, our analysis provides insights into how compounds in the MPDB align with Lipinski's Rule of Five. A notable 64% of the compounds fully meet the criteria, indicating they likely have properties conducive to oral drug administration. On the other hand, about 27% of the compounds register a single violation, suggesting a minor deviation from the ideal properties, but this doesn't automatically diminish their potential as drug candidates. A smaller fraction, around 3%, has two violations. Interestingly, while 5% of the compounds exhibit three violations, diverging from Lipinski's guidelines, they still present a structural framework that could inspire chemists to design improved drugs. This data is invaluable for drug discovery initiatives, guiding the prioritization of compounds based on their potential as orally administered drugs. The MPDB database stands out as a valuable asset for drug discovery, boasting a vast array of potentially druggable compounds. However, a deeper dive into the pharmacological and therapeutic potential of these compounds is warranted in subsequent research.

## Compound activities:

**Figure 6:**
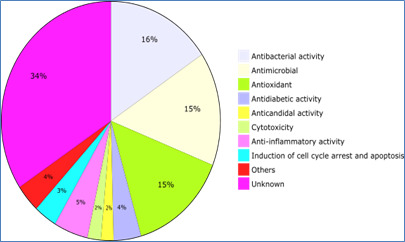
MPDB phytochemical biological activities. A diagram chart showing the distribution of various biological activities of a total of phytochemicals that have documented use in their references in MPDB.

In this study, we report on the distribution of biological activities for MPDB phytocompounds, as determined from the source reference. Our findings revealed that the most common biological activities observed were antibacterial (17.40%), antimicrobial (17.16%), and antioxidant (16.18%). In addition, anti-diabetic (4.53%), anti-candidal (2.08%), cytotoxicity (2.21%), anti-inflammatory (5.64%), and induction of cell cycle arrest and apoptosis (3.80%) activities were also observed. Other activities, accounting for 4.18%, include vaso-relaxant, anti-fungal, insecticidal, antiviral, analgesic, anti-proliferative, sedative effects, and psychoactive properties (Figure 6). Notably, our analysis highlights that 38.24% of the compound recordings lack information on their biological activities, underscoring the limited knowledge currently available on the phytochemical activity of these compounds. These findings emphasize the need for further investigation into the biological activities of phytocompounds and the potential implications for their use in medicinal applications.

## Analogs collection:

**Figure 7:**
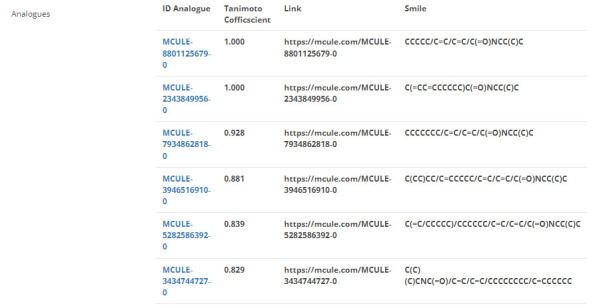
Snapshot of the analogue summary page for entry MNPDB00482.

In our comprehensive analysis, we successfully integrated a substantial number of analogs, totaling 65,700 analoges, into our dataset. Each of these analogs was accompanied by its corresponding SMILES notation, providing a standardized representation of the compound's structure. Additionally, direct links to the Mcule database were established for each analog, facilitating easy access to detailed compound information and further enhancing the utility of our dataset. To assess the structural similarity between the integrated analogs and the original compounds in the MPDB, we employed the Tanimoto coefficient (Figure 7). This metric provided insights into the degree of similarity, enabling us to gauge the relevance and potential applicability of the analogs concerning to the original phytocompounds. The inclusion of such a vast number of analogs, complete with SMILES notations, Mcule links, and Tanimoto coefficients, significantly enriches the MPDB, paving the way for more in-depth cheminformatics analyses and drug discovery endeavors in the future.

## Discussion:

The Moroccan Phytochemicals Database (MPDB) serves as a comprehensive resource for those interested in exploring the therapeutic potential of Moroccan plant phytochemicals. Research has shown that Moroccan plants offer a wide range of biological activities, including antimicrobial, anticancer, antidiabetic, and cardiovascular effects [14, 15,16,17]. The MPDB provides access to a carefully curated collection of phytochemicals derived from these plants, making it an essential tool for researchers in drug discovery, traditional medicine, and phytochemistry. The database contains information on over 700 unique compounds, categorized by their structural motifs. A notable segment of the database is dedicated to prenol lipids, which are celebrated for their diverse biological roles and therapeutic potential. These lipids play vital roles in cellular processes, such as maintaining cell membrane integrity, protein modification, and signal transduction [18]. One prominent example of prenol lipid, limonene, found abundantly in Ammodaucus leucotrichus, Citrus limon peel, and Origanum grosii [19], [20], [21], has been recognized for its chemopreventive and chemotherapeutic activities against various cancers. Research indicates that limonene induces apoptosis in cancer cells [22]. Moreover, limonene exhibits antimicrobial properties against a broad spectrum of bacterial and fungal pathogens [23] and possesses anti-inflammatory effects [24]. Flavonoids also feature prominently, making up 7.75% of the database. These polyphenolic compounds, commonly found in plants, are known for their therapeutic benefits. With a 15-carbon skeleton, they're further classified into subclasses like flavonols, flavones, and isoflavones [25].The significant presence of flavonoid-rich plants in the MPDB highlights the therapeutic importance of these compounds. Numerous studies have emphasized the antioxidant properties of flavonoids, attributing their health benefits to their capacity to neutralize free radicals and counteract oxidative stress [26]. Their anti-cancer, anti-inflammatory [27], and antimicrobial [28] effects are also well-established. Tropane alkaloids, representing 3.76% of the database, are distinguished by their unique tropane skeleton-a seven-membered ring structure containing nitrogen. These compounds are primarily found in Solanaceae family plants, such as Datura (Datura stramonium) and Henbane (Hyoscyamus albus). Noteworthy among these are Atropine [29], Scopolamine [30], and Hyoscyamine [31], they have significant pharmacological benefits, Atropine is primarily used to treat bradycardia and as a pre-anesthetic agent, while also serving as an antidote for cholinergic poisoning. Scopolamine is renowned for its efficacy against motion sickness and has potential roles in treating CNS disorders. Hyoscyamine offers relief from gastrointestinal and urinary disorders due to its antispasmodic properties. The database further showcases a variety of compounds. Fatty acyls, including the cardiovascular-beneficial Omega-3 fatty acids, constitute 3.29% [32]. Carboxylic Acids and their Derivatives, crucial in many biological processes, make up 1.88%, alicylic acid, in particular, is recognized for its anti-inflammatory properties [33]. Cannabinoids, accounting for 1.76%, have recently garnered significant attention for their potential therapeutic roles, especially in pain management and neuroprotection [34]. Morocco's recent decision to legalize cannabis for medical, cosmetic, and industrial uses not only acknowledges its therapeutic value but also sets the stage for its expanded global use. Argania spinosa, or the argan tree native to Morocco, is a treasure trove of bioactive compounds. Argan oil, extracted from its seeds, has been traditionally used for culinary, cosmetic, and medicinal applications [35]. Rich in unsaturated fatty acids like oleic and linoleic acid, argan oil is known for its anti-inflammatory and cardiovascular protective properties [36]. Additionally, argan oil boasts a rich content of tocopherols, notably gamma-tocopherol, which are renowned for their antioxidant properties that shield cells from oxidative damage [37]. The oil's polyphenol content further amplifies its antioxidant, anti-inflammatory, and anti-proliferative activities [38]. Unique to argan oil are the rare plant sterols, schottenol and spinasterol, which are postulated to have anti-inflammatory and potential anti-cancer benefits [39, 40,41,42]. The MPDB is meticulously designed to support computer-aided drug discovery techniques, such as molecular docking and virtual screening. The database's accurate 3D structures empower researchers to forecast potential therapeutic interactions with target proteins [43]. Virtual screening has notably risen as a cornerstone in drug discovery, facilitating the pinpointing of bioactive compounds from expansive databases and thus accelerating the drug development trajectory [44], [45]. Every piece of information within the MPDB is diligently sourced from scientific literature, providing a robust and trustworthy foundation for researchers. The fusion of virtual screening with age-old medicinal wisdom, as exemplified by the MPDB, heralds a transformative era in drug discovery, presenting a harmonized approach to unearthing new therapeutic agents.

## Conclusion:

The MPDB is a significant contribution to the field of drug discovery, particularly for researchers interested in the therapeutic potential of Moroccan plant chemicals. The database's manual curation from scientific literature ensures high-quality and reliable data, making it a valuable resource for in silico drug discovery efforts. The preliminary evaluations of phytochemicals against cardiovascular disease targets demonstrate the potential of MPDB to contribute to the identification of new therapeutic agents. Furthermore, the database supports the exploration of the scientific basis for the medicinal use of Moroccan plants, which have a rich tradition of use for therapeutic purposes.
